# Evaluation of the Blood Neutrophil-to-Lymphocyte Ratio (NLR) as a Diagnostic and Prognostic Biomarker in Dogs with Portosystemic Shunt

**DOI:** 10.3390/vetsci11020080

**Published:** 2024-02-09

**Authors:** Anja Becher, Els Acke, Gonçalo Serrano, Ingmar Kiefer, Michaele Alef, Wolf von Bomhard, Romy M. Heilmann

**Affiliations:** 1 Department for Small Animals, Veterinary Teaching Hospital, College of Veterinary Medicine, University of Leipzig, 04103 Leipzig, SN, Germany; becher.anja@gmx.de (A.B.); els-acke@idexx.com (E.A.); kiefer@kleintierklinik.uni-leipzig.de (I.K.); alef@kleintierklinik.uni-leipzig.de (M.A.); 2 IDEXX Vet Med Labor GmbH, 70806 Kornwestheim, BW, Germany; 3 Small Animal Department, Faculty of Veterinary Medicine, Ghent University, 9820 Merelbeke, Belgium; goncalo.serrano@ugent.be; 4 AniCura Haaglanden Specialist Referral Centre, 2288 EZ Rijswijk, The Netherlands; 5 Antech Specialty Center for Veterinary Pathology, 81477 Munich, BY, Germany; wolf.vonbomhard@synlab.com

**Keywords:** canine, hepatic encephalopathy, histopathology, hypoadrenocorticism, systemic inflammatory response, surgical ligation

## Abstract

**Simple Summary:**

Routine blood parameters (e.g., the neutrophil-to-lymphocyte ratio, NLR) are valuable tools for diagnosing inflammatory, infectious, and metabolic conditions. Dogs with congenital liver shunts show evidence of generalized inflammation and clinical signs that may overlap with those of other diseases. Therefore, the potential diagnostic and prognostic value of blood cell ratios, including the NLR, was investigated in 106 dogs with congenital liver shunts and compared to two other disease groups. An association was detected between the blood NLR and systemic inflammation but not with any characteristics of the liver shunt or clinical correlates. The NLR could not distinguish the three disease groups, dogs with liver shunts that were either medically or surgically treated, or dogs either with successful shunt surgery or those with surgical complications. However, lower NLRs (<2.53) were predictive of dogs with liver shunt surgery requiring only one PSS closure session rather than two consecutive surgeries. Thus, evaluating the blood NLR might be clinically useful in dogs with liver shunts. Although this parameter may have little value in distinguishing dogs with a liver shunt from dogs with other diseases, it appears to be predictive for the number of surgical sessions required for shunt closure via ligation.

**Abstract:**

The neutrophil-to-lymphocyte ratio (NLR) can help in assessing inflammatory diseases, sepsis, and chronic hepatic conditions in humans. Dogs with congenital portosystemic shunts (PSSs) have signs of generalized inflammation, and the clinical signs can overlap with other conditions, including hypoadrenocorticism (HOC). Thus, the potential diagnostic and prognostic value of leukocyte ratios as surrogate markers was assessed in a retrospective case–control study including 106 dogs diagnosed with PSSs. The disease control groups were dogs with parenchymal hepatopathy (PH; *n* = 22) or HOC (*n* = 31). In the PSS dogs, the blood NLRs were associated with the severity of systemic inflammation but not with the shunt type, hepatoencephalopathy, systemic infection, or hypoglycemia. The baseline NLRs did not differ between the three disease groups, between medically and surgically treated PSS dogs, or between those with successful PSS ligation and dogs experiencing peri-/post-surgical complications. However, dogs requiring two consecutive surgical interventions had significantly higher NLRs, and an NLR of <2.53 distinguished dogs with successful shunt ligation in one surgery from those requiring two consecutive surgeries for PSS closure. The blood NLR might be a useful clinicopathologic variable in PSS, but its value in helping differentiate PSS from HOC cases appears low. Integrating the NLR into a diagnostic algorithm may allow for a prediction of the number of surgical interventions required.

## 1. Introduction

Portosystemic shunts (PSSs) are acquired or congenital vascular anomalies that can occur in dogs and cats [1,2]. The clinical signs of a PSS result from impaired liver function due to the blood bypassing the liver, connecting the vena porta via an extrahepatic or intrahepatic shunting vessel to the systemic circulation [2]. With a prevalence of approximately 0.18% in dogs, PSSs are considered the most common congenital disorder of the hepatobiliary system [3]. Congenital extrahepatic shunts (ehPSSs) are more common in small breeds, while congenital intrahepatic shunt vessels dominate in larger breeds [3]. The clinical signs associated with PSSs may occur at any age and include gastrointestinal (GI), urological, and central nervous system signs [1]. Some cases of PSSs may initially resemble dogs with hypoadrenocorticism (HOC) as both groups of dogs can present with similar clinical signs and overlapping clinicopathologic findings.

The diagnostic work-up of dogs presenting with a suspected PSS includes routine blood work and measurement of the ammonia concentration, a serum bile acid stimulation test (SBA-ST), urinalysis, abdominal ultrasonography, computed tomography (CT), and/or arterial portography to evaluate the presence and location of the PSS and determine the possibility of surgical attenuation or closure [1,3,4]. In dogs that are not candidates for surgical intervention, conservative medical therapy is recommended, which was reported to result in a generally lower quality of life and a shorter median survival time compared to surgical treatment in a previous study [5]. However, surgical attenuation or closure carries potential peri- and post-operative risks, including irreversible neurological complications, the migration of occlusion devices, complications associated with portal hypertension, and death [6]. Histologic evaluation of liver biopsies has been previously shown to not serve as a prognostic marker for dogs undergoing surgical PSS attenuation [7]. However, several studies have investigated the potential utility of routine blood work, liver function tests, and other biomarkers to aid in the diagnosis and prognosis of chronic liver diseases, including PSSs, and in determining the surgical outcomes of PSS attenuation [8,9,10,11,12]. A proportion of 20% to 60% of dogs with severe hepatic dysfunction, including PSSs, develop hepatoencephalopathy (HE) [9,13,14], and high plasma ammonia concentrations are predictive of the presence of HE in humans and dogs [15,16]. Leukogram changes, including the absence of a clear stress leukogram, similar to dogs with typical or atypical hypoadrenocorticism (Addison’s disease), are anecdotally described in dogs with PSSs, but the diagnostic and/or prognostic value of leukogram changes in canine PSS patients and the differentiation of PSSs from HOC has not been reported.

The neutrophil-to-lymphocyte ratio (NLR) is a simple parameter to obtain within a routine diagnostic evaluation. In human medicine, the NLR is a useful marker for assessing inflammatory diseases, sepsis, and chronic hepatic conditions such as fibrosis and cirrhosis [10,12]. Thus, this study aimed to assess the potential diagnostic and prognostic value of the NLR and other leukocyte ratios as surrogate laboratory markers in dogs with PSSs. We hypothesized that the NLR predominantly correlates with the severity of HE, the SIRS score and/or presence of sepsis, the severity of histologic liver lesions, and the corresponding consequences of surgical intervention vs. medical treatment. Furthermore, we aimed to assess the utility of using the NLR to characterize PSSs in dogs and to aid in distinguishing dogs with PSSs from HOC cases.

## 2. Materials and Methods

### 2.1. Sampling Population

**Figure 1:**
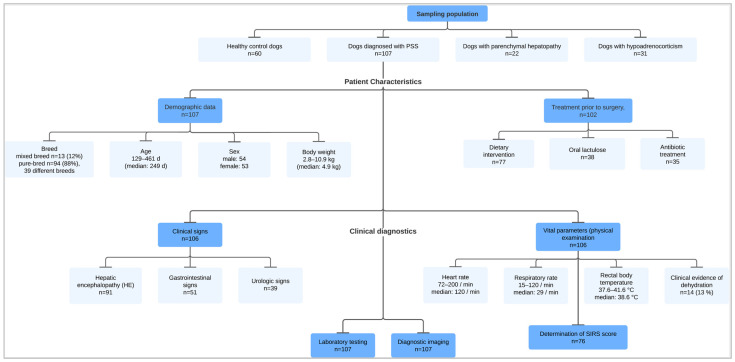
Study design and sampling population. This flowchart shows the groups of dogs included in this retrospective case–control study and the diagnostic evaluation of the dogs diagnosed with a portosystemic shunt (PSS).

This retrospective study included data from 107 dogs diagnosed with PSSs at the Department for Small Animals at the University of Leipzig (UL), Germany, from May 2011 to February 2022. For comparison of the data, two disease control groups of dogs diagnosed with either parenchymal hepatopathy (PH; *n* = 22) or hypoadrenocorticism (HOC; *n* = 31) and a healthy control group (*n* = 60) were also included (Figure 1). Medical data were extracted from the standard electronic patient medical records, which were either fully obtained by the attending veterinarian from the dog’s owner during consultation at the internal medicine service at the University of Leipzig College of Veterinary Medicine (UL)’s Department for Small Animals or completed using prior medical records provided by the referring veterinarian or clinic. Ethical review and approval, as well as informed owner consent for this retrospective investigation, were not required as the owners had provided written permission to use anonymized patient data for research purposes in the standard patient admission form used at the UL Department for Small Animals.

Dogs with PSSs—As an inclusion criterion for the PSS group, the diagnosis of a PSS had to be verified using either abdominal ultrasonography or contrast computed tomography (CT) scan. If surgical intervention following further diagnostics under general anesthesia (CT) was not elected by the owner, dogs with confirmatory ultrasonographic findings (visualization of a distinct shunting vessel) or a combination of strongly suggestive ultrasonographic changes (renomegaly or reno-splenomegaly, microhepatica, altered hepatic echotexture with a lack of clear portal vein ramification and/or turbulence in the bloodstream within the vena cava, and renal parenchymal mineralization and/or crystalloid debris or uroliths within the urinary bladder) allowing a PSS diagnosis based on the integration of all diagnostic features (consistent signalment and clinical signs, blood work results, and diagnostic imaging) [1,4] were also included.

The demographic data and medical history (including prior dietary intervention, lactulose, and antimicrobial treatment) were obtained from the owner and prior veterinary examinations as described in the patient records. According to the patient history, clinical signs were assigned into categories based on the primary organ system involved (i.e., neurologic, urologic, and/or GI signs). The clinical grade of HE was determined based on the HE grading scheme proposed by Anh et al., 2016 (Table 1) [17].

The vital parameters (heart rate, respiratory rate, rectal body temperature, and clinical evidence of dehydration) of the dogs at the first presentation were extracted from the patient records and used to evaluate evidence of systemic inflammation (SIRS) with or without infection (sepsis). SIRS was defined to be present if at least 2 of the 4 following criteria were fulfilled: tachycardia (>140/min), tachypnea (>40/min), hypothermia (<37.2 °C) or hyperthermia (>39.2 °C), leukocytosis (>19.5 × 10^9^/L), or leukopenia (<5.0 × 10^9^/L) [18]. This system was also used to calculate the SIRS score (Table 2). Dehydration was separately considered because dogs with clinically detectable (≥5%) dehydration can show similar vital parameters to SIRS dogs but without having systemic inflammation [19]. Clinical evidence of dehydration was defined to be present (and subcategorized as mild, moderate, or severe vs. euhydration) depending on the following findings extracted from the medical records of the patient’s physical examination at admission: reduced or lost skin elasticity, dry or tacky mucous membranes, thick sticky saliva, excessive panting, and, in severe cases, also enophthalmos, tachycardia, a weak pulse, a prolonged capillary refill time, and/or an altered mental status [20].

Dogs with PH—For dogs to be included in this group, liver tissue biopsies had to be obtained (laparoscopically or via laparotomy) for histopathology, bacterial culture, and/or quantitative copper analysis, following a histopathological diagnosis of parenchymal hepatopathy. Clinical signs (e.g., polyuria/polydipsia, lethargy, weight loss, vomiting, and/or diarrhea), laboratory findings (e.g., microcytic anemia, increased liver enzyme activities, hyperbilirubinemia, hypocholesterolemia, increased serum bile acids), testing on coagulation status, and/or abdominal diagnostic imaging findings consistent with the diagnosis of a parenchymal hepatopathy were also retrieved from the medical records and included in the analysis. Cholecystocentesis and the bile cytology results, when performed, were retrieved.

Dogs with HOC—For dogs to be included in this group, hypoadrenocorticism (atypical or typical) had to be confirmed using an ACTH stimulation test, and the administration of adrenosuppressive medications within at least 4 weeks had to be excluded. Clinical signs (e.g., polyuria/polydipsia, GI signs such as vomiting and/or diarrhea and severe lethargy or stupor when presenting in an Addisonian crisis) and laboratory findings (e.g., microcytosis with or without anemia, hypercalcemia, hypoglycemia, hypocholesterol-emia, azotemia, hyponatremia and hyperkalemia, a low baseline cortisol concentration) suggestive of a diagnosis of HOC [21,22] were retrieved from the medical records and included in the statistical analysis. 

Healthy controls—The control group consisted of healthy pet dogs enrolled in the blood donor program at the UL Department for Small Animals. Prior to inclusion, and regularly while enrolled in this program, a complete physical examination and blood work (complete blood cell count, serum biochemistry panel, determination of blood group, testing for relevant transmissible vector-borne diseases) served to confirm the overall healthy clinical status of these dogs.

### 2.2. Diagnostic Investigation

Whole blood was used for routine hematology performed using an IDEXX ProCyte (*n* = 199), Sysmex XN-9100 (*n* = 16), or LaserCyte (*n* = 1) at the Small Animal Veterinary Diagnostic Laboratory at the UL (*n* = 195) or externally (*n* = 25; IDEXX analyzer not documented for *n* = 4 dogs). The automated cell counts were confirmed using blood smear analysis (manual differentiation) in 9 cases. The neutrophil, lymphocyte, monocyte, and eosinophil counts were extracted from the hematology profiles; the NLR was calculated as [(neutrophil count)/(lymphocyte count)], the ELR (eosinophil-to-lymphocyte ratio) was calculated as [(eosinophil count)/(lymphocyte count)], the ENR (eosinophil-to-neutrophil ratio) was calculated as [(eosinophil count)/(neutrophil count)], and finally, the EMR (eosinophil-to-monocyte ratio) was calculated as [(eosinophil count)/(monocyte count)].

Serum samples were used for a biochemistry profile performed using a commercial analyzer (FUJI DRI-CHEM NX500i, Scil, for UL cases; *n* = 195), including the serum liver enzyme activities, and for the measurement of serum cortisol, pre- and post-prandial bile acid concentrations (SBA-ST), and ammonia (from heparin plasma or whole blood samples). The serum chemistry results were extracted from the referring veterinarian’s medical records in dogs lacking complete in-house laboratory testing at the UL (*n* = 25). 

Urine samples (*n* = 59) were used for routine urinalysis (performed at the Small Animal Veterinary Diagnostic Laboratory at the UL or externally), including urine specific gravity, dipstick assay, and sediment analysis, and, if indicated, for further diagnostic testing (e.g., quantitative urine culture to evaluate for bacterial infection).

Diagnostic imaging was performed at the UL Department for Small Animals and included abdominal ultrasonography, a contrast CT scan, and/or contrast portography to verify the presence of a PSS. Dogs were only included in the analysis if a suspected PSS could be confirmed (all surgically managed and some medically managed cases) or presumed given the results of the combined diagnostics (medically managed cases). Through reference to the CT scan and/or portography, PSSs were subcategorized as either intrahepatic or extrahepatic, and the abnormally connecting (shunting) abdominal vessels were identified whenever possible. The diameter of the shunting vessel, measured based on the CT or portography at the first surgical intervention, was also extracted from the patient record (if documented).

Depending on the dog’s general condition and overall health status, vital parameters, and laboratory findings, as well as the PSS location, diameter, and course of the shunting vessel, treatment was elected based on the owner’s informed decision. Patients were either placed on medical treatment (consisting of a combination of lactulose, antimicrobials, anticonvulsants, gastroprotective agents, antiemetics, and/or a special hepatic support diet) or underwent surgical shunt attenuation (ligation) in one or two sessions after stabilization via medical treatment. Suture ligation, which is only one possible method for the surgical occlusion of PSSs besides the use of ameroid constrictors, cellophane bands, or a gradual intravascular occlusion via coil embolization [23], was performed in all patients undergoing surgical shunt attenuation at the UL Department for Small Animals by a single surgeon who was highly experienced in PSS surgery (MA). The decision to ligate the shunt vessel in either one single or two consecutive sessions was at the discretion of the experienced surgeon (MA) and was based on intraoperative manometric assessment and/or visual inspection of the surgical situs for evidence of severe acute portal hypertension [3]. Shunt ligation was considered “successful” when the shunting vessel could be completely ligated in one or two sessions and complete remission or a significant reduction in clinical signs was seen until hospital discharge. The procedure was considered “unsuccessful” when the shunting vessel could not be partially (1st session) or completely (1st or 2nd session) ligated and/or peri- or post-procedural complications (e.g., seizures) occurred. In a few severely affected cases, humane euthanasia was elected by the owners, either directly at diagnosis or following unsuccessful medical treatment and surgical intervention being declined by the owner. Individual disease courses and outcomes, given the choice of treatment, were extracted from the medical records of patients presented for a follow-up consultation. However, a comparison of follow-up blood analysis was not possible given the small number of patients presenting at the referral institution for long-term rechecks.

Samples of liver tissue (wedge biopsies) were obtained from 24 dogs undergoing surgery, and routine histologic evaluation of these liver tissue biopsies was performed. The severity of hepatic fibrosis and lipid granulomas was retrospectively graded on a 3-point scale (0 = absent, 1 = mild lesions, 2 = moderate lesions, 3 = severe lesions) based on the histopathology report [24].

### 2.3. Statistical Analysis

A statistical software package (JMP v.13) was used for all statistical analyses. Continuous data were tested for normality of their distribution using a Shapiro–Wilk *W* test, and the summary statistics for these variables are reported as medians (ranges) and those for categorical data as counts (*n*) and percentages. A non-parametric Wilcoxon rank-sum test or Kruskal–Wallis test was used for two- or multiple-group comparisons (i.e., different groups of dogs or dichotomous variables such as medical and/or dietary treatments), and the non-parametric Spearman’s rank correlation coefficient rho (ρ) was used to assess for correlations among continuous (and ordinal) variables. Spearman’s ρ was interpreted as reflecting a very strong (0.8–1.0), strong (0.6–0.8), moderate (0.4–0.6), weak (0.2–0.4), or very weak (0–0.2) correlation. A receiver operating characteristic (ROC) curve analysis was used to calculate the sensitivity and specificity at the optimum cut-off concentrations (determined using Youden’s index) to differentiate subgroups of dogs with PSSs. Statistical significance was set at *p* < 0.05.

## 3. Results

### 3.1. Patient Clinical Data

The dogs with PSSs were significantly younger than the dogs in the other three groups (Table 3), with no differences among those other groups. No differences were seen in the sex distribution, but the body weights were the lowest and the proportion of purebred dogs was the highest in the PSS group (Table 3), representing 39 different dog breeds.

PSS group of dogs—Presenting clinical signs were recorded for 106 of the dogs in this group (Table 4) and comprised neurologic signs in 83 dogs (78%), followed by GI signs in 51 dogs (48%) and urologic signs in 39 dogs (37%), which included polyuria/polydipsia in 36 dogs (34%). Altogether, 91/106 dogs (86%) were determined to have hepatoencephalopathy (HE), with their HE scores ranging from 1 to 4 (median: 3); 11/102 dogs (11%) were hypoglycemic; and 4/68 dogs (6%) had evidence of systemic infection. The differential leukocyte counts and erythrocyte parameters are summarized in Table 3: the platelet counts ranged from 62 to 914 × 10^9^/L (median: 231 × 10^9^/L, *n* = 104). The clotting times were PT = 9–25 s (median: 14 s, *n* = 76; reference interval: <17 s) and aPTT = 66–300 s (median: 104 s, *n* = 76; reference interval: <102 s). The indirect serum markers of liver function and serum liver enzyme activities are summarized in Table 5. The baseline serum cortisol was 34.8–179.3 nmol/L (median: 44.3 nmol/L) in six dogs and was <55 nmol/L in four of these dogs (67%), one of which had an ACTH stimulation test performed to exclude atypical HOC (stimulated serum cortisol concentration of 455.4 nmol/L). Ammonium urate crystalluria was detected in 22 of 58 (38%) urine sediment analyses. Just 1 of the 10 urine cultures was positive, growing *Escherichia coli* and *Stenotrophomonas maltophilia*.

The prior medical treatment before subjecting the dogs to surgery for PSS closure (this excluded dogs in which an acquired shunt could not be definitively excluded) consisted of dietary intervention in 77/102 dogs (76%), lactulose in 38/102 dogs (37%), and antimicrobial treatment (including ampicillin, amoxicillin/clavulanic acid, metronidazole, marbofloxacin, enrofloxacin, cefquinome, and gentamycin) in 35/101 dogs (35%). The PSS was extrahepatic in 61 dogs (57%) and intrahepatic in 28 dogs (26%); in 18 dogs, these could not be distinguished. Differentiation between congenital and acquired PSSs could be made in 59 dogs, with the majority (56 dogs, 95%) having a congenital shunt, which was also suspected in the remaining undifferentiated 48 dogs. The minimum shunt diameter (recorded for 63 dogs) ranged from 1.5 to 20 mm (median: 6 mm). The liver biopsies revealed fibrosis in 7/24 dogs (29%), with severity scores ranging from 1 to 2 (median: 2), and hepatic lipid granulomas were detected in 8/24 dogs (33%), with severity scores of 1–2 (median: 1.5). Medical treatment only was elected in 34 dogs (32%), whereas in 57 dogs (53%), the PSS was closed via ligation in two procedures (*n* = 37) or one procedure (*n* = 15). The PSS could not be completely ligated in 15 dogs (14%), and euthanasia was elected after diagnosis in 6 dogs (6%).

PH group of dogs—This group included dogs diagnosed with chronic hepatitis (*n* = 10), suppurative or reactive hepatitis (each *n* = 3), hepatic cirrhosis or primary portal vein hypoplasia (microvascular dysplasia, each *n* = 2), and marked nodular hyperplasia or hepatocellular carcinoma (*n* = 1). The clinical presentation, recorded for 21 dogs in this group (Table 4), included GI signs in 11 dogs (52%), followed by neurologic signs in 2 dogs (10%, with 1 dog determined to have HE) and urologic signs in 1 dog (5%). The serum biochemistry results are summarized in Table 5. One dog (1/17, 6%) was hypoglycemic, and one dog had evidence of systemic infection. The PT was 11–24 s (median: 14 s, *n* = 19), the aPTT was 79–300 s (median: 100 s, *n* = 18), and the platelet counts were 31–832 × 10^9^/L (median: 194 × 10^9^/L, *n* = 19). The liver biopsies showed fibrosis in 14 dogs (64%), with severity scores ranging from 1 to 3 (median: 2), and hepatic lipid granulomas in 4 dogs (18%). 

HOC group of dogs—This group comprised 27 dogs (87%) with typical HOC and 4 dogs (13%) with atypical HOC. The liver function markers (Table 5), except for cholesterol, glucose, and bilirubin, in dogs with HOC differed significantly from those in dogs with PSSs.

### 3.2. Comparison of Clinicopathologic Parameters between Groups

**Figure 2:**
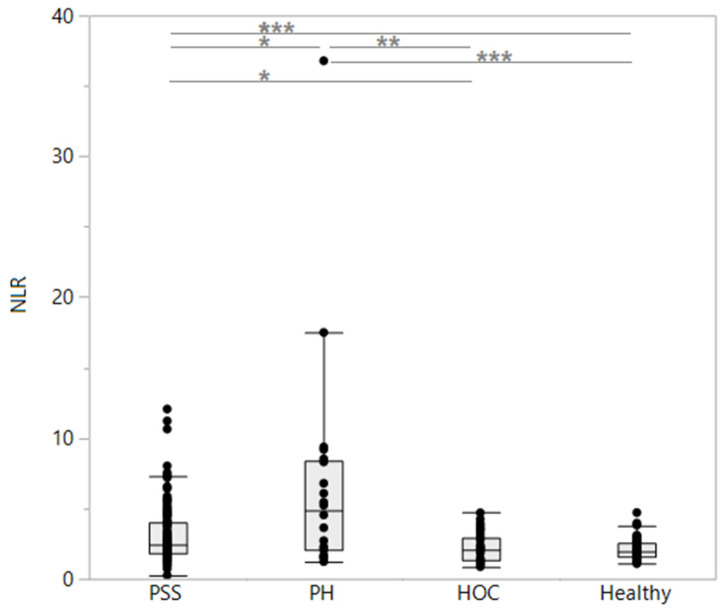
Neutrophil-to-lymphocyte ratios (NLRs) in dogs included in the study (*n* = 220). Compared to dogs with portosystemic shunts (PSSs, *n* = 107), the NLR was significantly higher in dogs with parenchymal hepatopathy (PH, *n* = 22; *p* = 0.0214) and significantly lower in dogs with hypoadrenocorticism (HOC, *n* = 31; *p* = 0.0217) and healthy control dogs (*n* = 60; *p* = 0.0007). However, a large overlap in the NLR is seen among all four groups of dogs. * *p* < 0.05, ** *p* < 0.01, *** *p* < 0.001.

The leukocyte and neutrophil counts were significantly higher in all disease groups of dogs compared to the healthy controls (Table 3). The lymphocyte counts were highest in the dogs with PSSs or HOC, and the monocyte counts were highest in the dogs with PSSs, followed by the dogs in the PH group. The lymphocyte and eosinophil counts were significantly higher in the dogs with PSSs than those diagnosed with PH but did not differ from the HOC dogs or the controls. The NLR was significantly higher in the PSS dogs compared to the HOC and control dogs but lower than those with PH, and a large overlap in the NLR was seen among all four groups of dogs (Figure 2). The ENR and EMR were intermediate in the dogs with PSSs and differed significantly between the dogs with PSSs and all other groups (Table 3). Hematology revealed the lowest erythrocyte parameters (hematocrit, MCV, MCH, MCHC, and Retic-HgB) in the PSS group of dogs, with a significantly lower MCV and MCH in the PSS dogs compared to all other groups.

### 3.3. Association of Leukocyte Ratios with Patient and Clinicopathologic Parameters in PSS Dogs

Sex, the shunt type (extrahepatic, intrahepatic, undetermined), and prior antimicrobial or lactulose treatment did not affect any of the leukocyte ratios (NLR, ELR, ENR, or EMR; all *p* > 0.05), but the ELR, ENR, and EMR were significantly higher in dogs receiving a liver support diet with or without additional hepatoprotectants (*p* = 0.0146, *p* = 0.0256, and *p* = 0.0019). Presentation with GI signs, neurological signs, urinary signs (including polyuria/polydipsia), signs of HE, evidence of systemic infection, and hypoglycemia were also not associated with the NLR at presentation (all *p* > 0.05).

The blood NLRs were moderately correlated with the SIRS scores, including (ρ = 0.37; *p* = 0.0009) or excluding hypoglycemia (ρ = 0.30; *p* = 0.0097), and with the total bilirubin concentrations (ρ = 0.36; *p* = 0.0081). Higher NLRs were weakly correlated with higher hematocrits (ρ = 0.21; *p* = 0.0355), serum BUN (ρ = 0.20; *p* = 0.0421), total protein (ρ = 0.22; *p* = 0.0265), and albumin concentrations (ρ = 0.22; *p* = 0.0265) and with lower eosinophil counts (ρ = −0.22; *p* = 0.0260) and serum ALP activities (ρ = −0.22; *p* = 0.0254). Higher serum BUN concentrations were also weakly correlated with a lower ENR (ρ = −0.25; *p* = 0.0113), ELR (ρ = −0.23; *p* = 0.0213), and EMR (ρ = −0.31; *p* = 0.0015).

### 3.4. Association of Leukocyte Ratios with Histopathology, Treatment, and Surgical Outcomes in PSS Dogs

**Figure 3:**
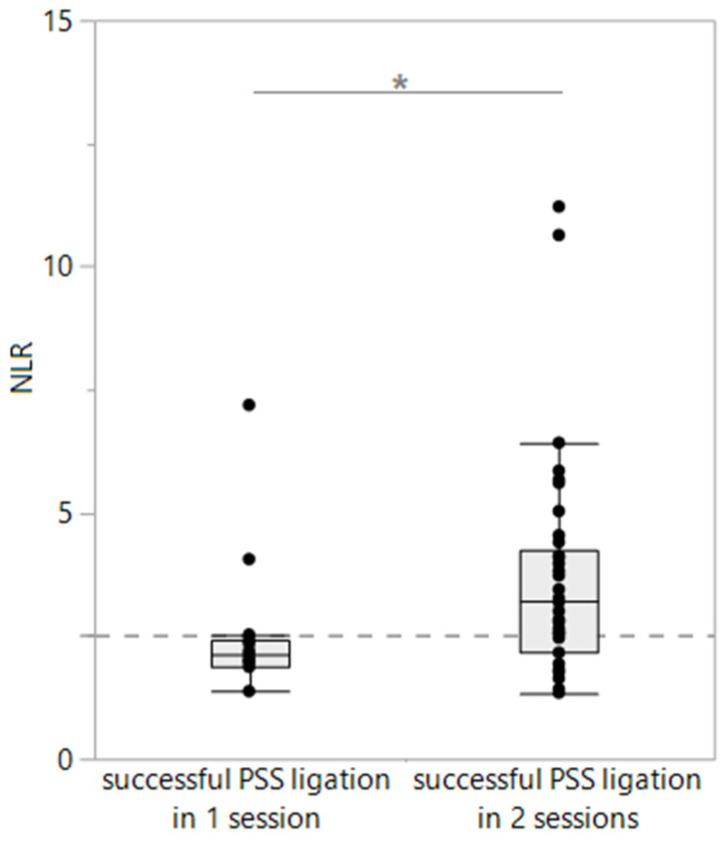
Neutrophil-to-lymphocyte ratios (NLRs) in dogs with successful surgical ligation of the portosystemic shunts (PSSs; *n* = 52). Blood NLRs were significantly higher in dogs requiring a two-step surgical approach to PSS ligation (*n* = 37) than in dogs with successful PSS ligation in one surgery (*n* = 15; *p* = 0.0213). An NLR of <2.53 (dashed vertical gray line) best distinguished dogs with successful one-step PSS ligation from those requiring two consecutive surgical interventions. * *p* < 0.05.

Comparing the blood NLRs at the time of presentation, no difference in the NLRs was seen between conservatively (medically, *n* = 31) and surgically treated dogs with PSSs (*n* = 67; *p* = 0.3169) nor between dogs with successful PSS ligation (*n* = 52) and those experiencing peri- or post-surgical complications (*n* = 15; *p* = 0.2318). The NLR did also not reflect the presence or severity of fibrosis (*p* = 0.3739 and *p* = 0.4239) or lipogranulomas (*p* = 0.7829 and *p* = 0.9306) in the liver histopathology (*n* = 24), which was similar for the blood ENR, ELR, and EMR (all *p* > 0.05). The pre- and post-prandial serum bile acid concentrations were strongly correlated with the severity of hepatic lipogranulomas (ρ = 0.61, *p* = 0.0071 and ρ = 0.62, *p* = 0.0104), as was the serum ALT activity (ρ = 0.53, *p* = 0.0072). Among the dogs with successful surgery, the blood NLRs were significantly lower in dogs with successful PSS ligation after just one intervention (median: 2.41; *n* = 15) than in dogs requiring a staged approach to PSS ligation with two consecutive surgical sessions (median: 3.19; *n* = 37; *p* = 0.0213; Figure 3). No such associations were seen for the ENR, ELR, or EMR. An NLR of <2.53 distinguished dogs with successfully ligated PSSs in just one surgery from dogs requiring two consecutive surgical interventions with 87% sensitivity (95% confidence interval [CI]: 62–98%) and 68% specificity (95% CI: 52–80%; area under the ROC: 71%).

## 4. Discussion

This study evaluated the potential utility of the blood NLR as a diagnostic and prognostic marker in dogs with PSSs, with the hypothesis that the NLR correlates with the severity of HE, the SIRS score and/or the presence of sepsis, the severity of histologic liver lesions, the decision on surgical intervention vs. medical treatment, and the surgical outcomes.

An important aim of the study was to investigate the utility of using the NLR to distinguish PSSs from cases with possible similar clinical presentation and/or overlapping clinicopathologic findings. Dogs with PSSs and those with HOC can show somewhat overlapping clinical signs, such as lethargy, polyuria/polydipsia, GI signs (e.g., vomiting, diarrhea), and a lean or poor body condition. The clinicopathological similarities include hypoglycemia, hypocholesterolemia, and the lack of a clear stress leukogram despite variable baseline serum cortisol concentrations [25,26]. The latter is likely due to a dysregulated renin–angiotensin–aldosterone system (RAAS) resulting from a lack of negative feedback [27] or critical illness-related corticosteroid insufficiency (CIRCI) [28] with severe clinical presentations of PSSs. Thus, a readily available parameter that could further aid in differentiating these conditions would be a very useful addition to the diagnostic repertoire in clinical practice. While the blood NLR might be a useful clinicopathologic variable in PSSs, the results of the study show that its value in helping differentiate PSS cases from HOC cases is rather low. In contrast, the dogs with PH had significantly higher NLRs and lower lymphocyte and eosinophil counts than the PSS cases, but other parameters and clinical presentation can help determine PSS cases from PH cases.

Analysis of the surgical liver biopsies revealed histopathological lesions that are commonly detected in dogs with PSSs, including lobular atrophy, portal vein collapse/hypoplasia, increased numbers of arterial cross-sections, lipid granulomas, and various grades of liver fibrosis [29,30]. A minimally invasive biomarker that reflects the extent of chronic remodeling might be a useful addition to an algorithm for clinical decision-making, but the blood NLR does not appear to serve this purpose based on the results of our study. However, the NLR was predictive of PSS attenuation being successful in one surgery rather than two surgical sessions, which is consistent with lower total white blood cell counts being associated with short-term survival in other studies [31,32]. 

Predicting the likelihood of a positive outcome is important when electing surgical vs. medical treatment for an individual patient with a PSS. The results of our study indicate that the NLR has no additive value in predicting the success of conservative (medical) vs. surgical treatment in dogs with PSSs nor in predicting the probability of peri- and/or post-surgical complications. In line with this, the blood NLR did also not reflect the presence or severity of hepatic (non-cirrhotic) fibrosis or lipogranulomas in routine liver histopathology, as was seen for the serum SBA-ST and ALT activity with the severity of hepatic lipogranulomas. However, this relationship warrants further systematic investigation with a larger population of PSS dogs, as liver tissue specimens were obtained in only 24 of the PSS dogs that underwent surgery. Lipid granulomas, which were significantly associated with the pre- and post-prandial serum bile acid concentrations and serum ALT activities, are formed by macrophages ingesting fat from ruptured lipid-containing hepatocytes [33], and their association with higher serum bile acid concentrations might reflect a more compromised liver function, potentially resulting from liver cell destruction, as reflected by the strong correlation with the serum ALT activities.

The success of surgical treatment and the individual patient outcomes are largely dependent on several predetermining and interacting factors, including the type, location, and diameter of the shunt; the type and severity of the corresponding clinical signs; the surgical approach and the workspace available in the surgical situs during surgery; the presence and extent of hepatic tissue remodeling; the timing of presentation and clinical condition of the dog at presentation; and the compliance of the owner and dog [7]. The blood NLRs were significantly higher in dogs with successful PSS ligation in two consecutive sessions than in dogs where the shunting vessel could be completely ligated in just one surgical intervention. The presence of clinical signs at surgery is associated with more surgical complications and systemic inflammation [9], and leukocytosis has been linked to a reduced short-term mortality after PSS closure but variable long-term outcomes [31,32,34]. However, a long-term follow-up could not be evaluated in this study.

The clinical signs predominating in PSS dogs—signs of HE, systemic infection, and hypoglycemia—were also not significantly associated with the blood NLR. Previous studies demonstrated that C-reactive protein (CRP) and ammonia are increased in dogs with clinical signs secondary to congenital PSSs [9,16]. Given the mild association between the NLR and SIRS scores (irrespective of hypoglycemia), the lack of a correlation of the NLR with HE presence and blood ammonia levels is surprising. However, the presence of HE and the severity of hyperammonemia can be affected by other variables (e.g., food intake, GI hemorrhage, and/or sample processing), which could be a possible explanation, and this information was not available due to the retrospective study design based on pre-existing patient medical records.

The results of this study confirm many previously reported aspects of PSSs in dogs. This includes that many dogs with PSSs in our study were purebred dogs, breeds commonly over-represented in studies on PSSs (e.g., Bolonka Zwetna, Yorkshire Terrier, Malteser, and Labrador Retriever), and of very young age [1,2,4]. However, some dogs included in our study had a later presentation, supporting the idea that the onset of clinical signs may occur at any age [1]. The majority of the dogs were diagnosed with an extrahepatic PSS, a more common finding in smaller breeds [35], which comprised the majority of our study population. Agreeing with many reports, most dogs had a typical presentation, including neurological (at 78% being the most common sign in our study), GI, and urological signs.

The clinicopathological results also confirmed the findings of others [1,4] in that many dogs had increased serum liver enzyme activities and pre- and post-prandial bile acid concentrations (SBA-ST), decreased plasma BUN concentrations, hyperammonemia, and ammonium urate crystalluria on urine sediment analysis. The blood leukocyte and neutrophil counts were significantly higher in all disease groups of dogs compared to the healthy controls, which confirms that a PSS is associated with a systemic inflammatory response [9]. This response has been proposed to be protective against the translocated bacteria in the portal blood [36].

The ELR, ENR, and EMR were significantly higher in dogs receiving a liver support diet with or without additional hepatoprotectants, which could reflect the eosinophil healing period and recovery of some liver tissue damage at the cellular level. Eosinophils are immunologically active cells that release biologically active lipids, vasoactive substances, and inflammatory as well as fibrosis-promoting mediators. However, a medication-elicited response might be an alternative explanation for the differences in the blood eosinophil ratios. The hematology findings in our study are also consistent with other investigations, revealing the lowest erythrocyte parameters (MCV and MCH) in the PSS group of dogs. Microcytosis and hypochromia are morphological erythrocyte changes that suggest iron deficiency (anemia), a common finding in dogs with PSSs [37,38]. The reticulocyte hemoglobin content (Retic-HgB) was also lowest in the PSS dogs and might be another important marker in this context, as it reflects the availability of iron for hematopoiesis and is not affected by the acute-phase response [39]. Retic-HgB might be decreased due to blood loss, inflammation (i.e., anemia of chronic inflammatory disease causing iron sequestration), a combination of both, reduced iron uptake from the intestine, and/or a reduced systemic iron-binding capacity, as well as in healthy immature animals [38,40,41]. Because of the short period of reticulocyte circulation, Ret-HgB may serve as an early and sensitive indicator of decreased iron availability due to inflammation, which might be seen in patients with PSSs [42].

An interesting finding is the lack of the effect of prior antimicrobial [18] or lactulose treatment on any of the leukocyte ratios given that the severity of HE has been shown to correlate with systemic inflammation in dogs with congenital PSSs [9]. In addition, a correlation of the serum CRP with the clinical score has been previously shown in dogs with medically managed congenital extrahepatic PSSs [16]. A possible explanation could be that some of these medications act primarily locally in the GI tract and have little effect on the systemic inflammatory response in dogs with PSSs (i.e., the inflammatory leukogram). Administration of lactulose mainly accelerates the intestinal passage and acidifies the lumen of the distal intestinal segments (colon), resulting in an unfavorable environment for certain toxin-producing bacteria with a net effect of reduced ammonia reabsorption (intestinal ammonia trapping). The antibiotics previously used as part of the treatment plan in dogs with PSSs also modulate the intestinal microbiome, creating a milieu that potentiates luminal ammonia trapping, and—in some dogs—addresses concurrent lower urinary tract infections (UTI) if verified based on an active urine sediment and/or bacterial culture. The antibiotics used for the last indication were those recommended for treating uncomplicated UTIs (e.g., amoxicillin/clavulanic acid) [43]. In PSSs, urate urolithiasis and a poor urine-concentrating ability can predispose dogs to UTIs [44,45]. 

Higher hematocrits and higher serum BUN, total protein, and albumin concentrations were mildly correlated with the NLR, all serving as indicators of dehydration or hypovolemia. Thus, careful interpretation of the correlation between the NLR and SIRS scores is warranted because the clinical signs of dehydration largely overlap with the clinical parameters in dogs with non-infectious SIRS [19]. Many dogs included in the study were presented for stabilization and diagnostic investigation when clinically markedly affected and in poor overall condition, presumably associated with significant systemic stress. With leukocytosis (>19.5 × 10^9^/L) being one criterion used to confirm SIRS and with neutrophilia with lymphopenia (resulting in an NLR increase) being part of the stress leukogram, the correlation between the NLR and SIRS is, to some extent, expected.

We acknowledge some limitations of the study. First, given the study’s retrospective design, there is a risk of some details being omitted in the patient medical records, which might have affected the accuracy of the retrospective subclassification of patients (e.g., the presence of SIRS or grade of HE). In line with this, the risk of bias in the authors’ assessment of individual patient parameters cannot be excluded given that the study’s retrospective nature dictated reliance on pre-existing information and precluded a direct examination of the dogs included. Second, the retrospective observation period of the study (2011–2022) spanned a relatively long time (11 years), resulting in some parameters (e.g., Ret-HgB) being missing for patients that were initially presented during the initial years of the observation period when this parameter was not available yet. Further, the data and bloodwork findings extracted from the patient records in this study showed that the severity and duration of the clinical signs, the prior treatment, and the individual timeline of the diagnostic evaluation and therapeutic intervention varied between the dogs with PSSs. The automated cell counts were confirmed using blood smear analysis (manual differentiation) in only nine cases. In addition, two-thirds of the medically managed dogs with (presumed) PSSs did not undergo more invasive diagnostics (i.e., CT portography, which is considered the gold standard for a diagnosis of a PSS) after weighing the increased risk of general anesthesia and radiation exposure against the lack of benefit given the predetermined choice of treatment elected by the owner. Compared to CT portography, the sensitivity and specificity of ultrasonography for PSS detection range from 68 to 95% and 67 to 100% [3], with a distinction between congenital intrahepatic and extrahepatic PSSs in 92% of cases and a generally higher sensitivity in detecting intrahepatic PSSs (95–100%) [3]. However, dogs with a presumptive PSS diagnosis (i.e., without laparotomy and/or CT portography) were only included in the study if their signalment, patient history, clinical signs (including those of HE), physical examination, and routine diagnostic findings (including SBA-ST and/or ammonia testing), together with abdominal ultrasonography, rendered a PSS the most likely diagnosis. The serum protein C concentration, for PSS diagnosis [8], and cobalamin (vitamin B_12_) and methylmalonic acid (MMA) concentrations, used to rule out congenital cobalamin deficiency, which can mimic a PSS [46], were not available for most of the PSS dogs in this study. Cobalamin deficiency can lead to an accumulation of MMA and—through the inhibition of the enzyme carbamoyl phosphate synthetase I, which normally metabolizes ammonia into carbamoyl phosphate—increased plasma ammonia concentrations [47]. However, hypocobalaminemia as a primary cause of hyperammonemia appears to be rare in dogs [47] and as a cause of clinical signs resembling PSSs was very unlikely in the dogs in this study given that PSSs were verified using CT and/or abdominal ultrasonography. 

Furthermore, differences in age and body weight between the PSS cases and the disease control (PH) group are to be expected given that predominantly younger dogs present with PSSs, whereas most inflammatory, degenerative, or neoplastic hepatopathies (PH) are diagnosed in middle-aged to older dogs. While age-related variations in the hematologic and biochemical variables might have contributed to the differences detected in this study (e.g., physiologically lower RBC counts and albumin and globulin concentrations and higher serum ALP activities during growth in young dogs), many variables differing between the PSS and PH groups (e.g., total white blood cell, lymphocyte, neutrophil, monocyte, eosinophil, and platelet counts; AST and ALT activity; BUN and bile acid concentrations) are not linked to age [48]. In addition, the PH group of dogs comprised several disease phenotypes, rendering it less than ideal as a disease control group. Lastly, liver tissue biopsies were not consistently obtained from all dogs that underwent surgical PSS attenuation via ligation. This led to small group sizes for further stratification and analysis of the potential correlations or associations between blood cell counts or ratios and the presence and severity of histologic lesions.

## 5. Conclusions

The blood NLR appears to have limited clinical utility as a diagnostic or prognostic marker in dogs with PSSs. It does not appear to provide additive information for selecting the most suitable therapeutic option for dogs with PSSs, disproving our primary hypothesis. Furthermore, the blood NLR was shown not to aid in the differentiation of dogs with PSSs from dogs diagnosed with HOC before confirming the diagnosis using additional endocrine testing (i.e., the ACTH stimulation test), particularly for atypical presentations lacking or compensating for mineralocorticoid deficiency. However, the group of atypical HOC cases was too small (*n* = 4) for further subgroup comparisons. Thus, the diagnosis, treatment, and prognosis of PSSs continue to rely on the integration of the results of several diagnostical tests, including routine blood work, urinalysis, diagnostic imaging, further diagnostics to rule out other differentials, and potentially hepatic biopsy, to arrive at the final diagnosis and optimize treatment for individual patients. However, integrating the NLR into a diagnostic algorithm, similar to that recently described for the diagnosis of HOC [49], may allow for a prediction of the number of surgical interventions required if shunt attenuation using surgical ligation is selected, but further prospective studies are needed to confirm our results. 

## Figures and Tables

**Table 1:** Hepatoencephalopathy (HE) severity grading scheme as proposed by Anh et al. [17].

Grade of HE	Clinical Signs
0	none (no clinical signs, unremarkable physical examination)
I	mildly decreased activity, lethargy, or both
II	severe lethargy, mild ataxia, or both
III	hypersalivation, severe ataxia, head pressing, blindness, circling, or a combination of these
IV	seizures and stupor or coma

**Table 2:** Calculation of the SIRS score as proposed by Singer et al. [18].

SIRS Score (Out of 4)	Leukocyte Count	Suspected Band Neutrophils	Heart Rate	Respiratory Rate
0	5.0–19.5 × 10^9^/L	no	<140/min	<40/min
1	Fulfilling 1 of 4 criteria: tachycardia (>140/min), tachypnea (>40/min), hypothermia (<37.2 °C) or hyperthermia (>39.2 °C), leukocytosis (>19.5 × 10^9^/L), or leukopenia (<5.0 × 10^9^/L)			
2	Fulfilling 2 of the 4 criteria		SIRS was defined to be present if at least 2 out of the 4 following criteria were fulfilled	
3	Fulfilling 3 of the 4 criteria			
4	Fulfilling all 4 criteria data			

**Table 3:** Patient characteristics and hematologic parameters in 220 dogs with either a portosystemic shunt (PSS), parenchymal hepatopathy (PH), or hypoadrenocorticism (HOC) or without evidence of disease included in the study. Summary statistics presented are medians (interquartile ranges) or counts (percentages).

Patient Characteristic	PSS	PH	HOC	Controls	*p*
*n*	107	22	31	60	–
**Patient characteristics**					
Age in days	**249 (129–461) ^A^**	2249 (978–4213) ^B^	1677 (1013–2433) ^B^	1551 (897–2449) ^B^	**<0.0001**
Age in years	**0.7 (0.4–1.3) ^A^**	6.2 (2.7–11.5) ^B^	4.6 (2.8–6.7) ^B^	6.5 (4.2–6.7) ^B^	
Male/female sex	54 (50%)/53 (50%)	8 (36%)/14 (64%)	18 (58%)/13 (42%)	34 (57%)/26 (43%)	0.3539
Body weight in kg	4.9 (2.8–10.9) ^A^	12.2 (8.0–18.2) ^B^	20.3 (8.5–25.5) ^B^	29.8 (26.5–34.0) ^C^	**<0.0001**
Breed					**0.0003**
- Purebred	**94 (88%) ^A^**	13 (59%) ^B^	22 (71%) ^B^	37 (62%) ^B^	
- Mixed breed	13 (12%)	9 (41%)	9 (29%)	23 (38%)	
**Clinicopathologic parameters**					
**Leukogram**					
Leukocyte count in ×10^9^/L	14.38 (10.97–19.16) ^A,^*	16.55 (9.36–24.23) ^A^	14.05 (10.97–17.71) ^A^	8.87 (7.26–10.48) ^B^	**<0.0001**
Neutrophil count in ×10^9^/L	8.50 (6.43–11.51) ^A^	11.94 (5.58–18.50) ^A^	8.03 (6.01–10.54) ^A^	4.78 (4.06–6.54) ^B^	**<0.0001**
Lymphocyte count in ×10^9^/L	3.28 (2.59–4.19) ^A^	2.24 (1.58–3.29) ^B^	3.74 (2.87–5.78) ^A^	2.53 (1.95–3.31) ^B^	**<0.0001**
Monocyte count in ×10^9^/L	1.37 (1.02–1.96) ^A^	1.10 (0.51–2.52) ^A^	0.67 (0.49–1.01) ^B^	0.51 (0.41–0.69) ^C^	**<0.0001**
Eosinophil count in ×10^9^/L	0.52 (0.34–0.93) ^A^	0.24 (0.09–0.47) ^B^	0.69 (0.41–1.10) ^A^	0.61 (0.44–0.84) ^A^	**<0.0001**
NLR	2.45 (1.86–4.05) ^A^	4.88 (2.06–8.35) ^B^	2.10 (1.31–2.95) ^C^	1.99 (1.60–2.52) ^C^	**<0.0001**
NLR < 2.3	47 (44%)	8 (36%)	20 (65%)	39 (65%)	**0.0113**
ELR × 10^2^	17.26 (9.58–24.86) ^A^	11.61 (3.66–18.47) ^B^	19.42 (11.48–29.31) ^A^	24.01 (17.05–37.11) ^C^	**<0.0001**
ENR × 10^2^	**6.28 (3.45–10.69) ^A^**	2.26 (0.64–7.24) ^B^	9.81 (6.13–12.43) ^C^	11.99 (9.37–15.49) ^D^	**<0.0001**
EMR × 10^2^	**45.59 (21.02–67.88)** ^A^	18.62 (7.04–58.46) ^B^	97.62 (72.41–132.7) ^C^	116.59 (85.62–153.6) ^C^	**<0.0001**
**Erythrogram**					
Hematocrit in %	36.6 (31.9–41.8) ^A,^*	43.2 (31.0–48.8) ^A,B^	43.8 (37.6–52.6) ^B,C^	47.2 (43.5–50.9) ^C^	**<0.0001**
MCV in fL	**56.5 (53.7–60.3) ^A^**	64.3 (61.6–69.7) ^B^	62.1 (59.8–63.2) ^C^	65.5 (63.8–67.2) ^B,D^	**<0.0001**
MCH in pg	**19.8 (18.9–21.0) ^A^**	22.3 (21.9–23.5) ^B^	23.0 (22.0–23.8) ^B,C^	23.3 (22.9–23.9) ^C^	**<0.0001**
MCHC in g/dL	35.4 (34.2–36.4) ^A,^*	35.6 (34.2–36.3) ^A,B^	37.3 (36.0–38.3) ^C^	35.8 (35.2–36.3) ^B^	**<0.0001**
Retic-HgB in pg	22.0 (21.0–24.8) ^A,$^	23.6 (22.2–26.2) ^A,B,†^	25.4 (22.8–27.4) ^B,‡^	27.2 (25.1–27.8) ^C,#^	**<0.0001**

ELR: eosinophil/lymphocyte ratio; ENR: eosinophil/neutrophil ratio; NLR: neutrophil/lymphocyte ratio; MCV: mean corpuscular volume; MCH: mean corpuscular hemoglobin; MCHC: mean corpuscular hemoglobin concentration; Retic-HgB: reticulocyte hemoglobin content. Values in bold font are significantly different between PSSs and all other groups of dogs. Values within the same row and not sharing a common superscript letter are significantly different at *p* < 0.05. * Available from *n* = 106 dogs; ^$^ available from *n* = 36 dogs; ^†^ available from *n* = 9 dogs; ^‡^ available from *n* = 14 dogs; ^#^ available from *n* = 18 dogs.

**Table 4:** Patient vital parameters and serum bile acid concentrations in dogs (*n* = 127) with either a portosystemic shunt (PSS) or parenchymal hepatopathy (PH) included in the study.

	PSS Group (*n* = 106)			PH Group (*n* = 21)			*p*
Parameter	Meas	Median or *n* (%)	Range	Meas	Median or *n* (%)	Range	
Cardiac rate (/min)	99	120	72–200	21	120	78–168	0.8350
Respiratory rate (/min)	79	29	15–120	15	32	18–52	0.4338
Clinical evidence of dehydration	105	14 (13%)	–	20	6 (30%)	–	0.3136
Rectal body temperature (°C)	85	38.6	37.6–41.6	15	38.5	38.1–40.0	0.5706
Increased body temperature	85	3 (4%)	–	15	2 (13%)	–	0.1609
Suspected band neutrophils	103	**10 (10%)**	–	15	**5 (33%)**	–	**0.0232**
SIRS score							
including hypoglycemia	76	0	0–3	13	0	0–4	0.8475
not including hypoglycemia	76	0	0–4	13	0	0–4	0.5356
Pre-prandial serum bile acid concentration (µmol/L)	87	73.2	0.2–527.1	6	71.1	4.2–167.1	0.4206
Post-prandial serum bile acid concentration (µmol/L)	70	**134.1**	**20.8–580.0**	3	**80.5**	**8.0–120.4**	**0.0469**
Difference in post- vs. pre-prandial serum bile acids	69	46.5	−159–352.8	3	5.0	3.4–53.7	0.3979

Meas: number of dogs in which the parameter was measured and documented. Values in bold font are significantly different between PSSs and the PH group of dogs.

**Table 5:** Serum indicators of liver function in dogs (*n* = 158) with either a portosystemic shunt (PSS), parenchymal hepatopathy (PH), or hypoadrenocorticism (HOC) included in the study.

	PSS Group (*n* = 106)			PH Group (*n* = 21)			HOC Group (*n* = 31)			*p*
Serum Parameter	*n*	Median	Range	*n*	Median	Range	*n*	Median	Range	
BUN (mmol/L)	102	2.7	0.8–21.2	18	5.7	1.8–21.0	31	21.5	6.2–72.8	**<0.0001**
Albumin (g/L)	107	26	10– 36	22	31	16–45	30	32.5	22–48	**<0.0001**
Total protein (g/L)	104	52	30–69	21	62	41–96	31	66	34–84	**<0.0001**
Cholesterol (mmol/L)	48	3.53	1.12–9.93	14	7.36	0.98–12.0	14	4.44	1.97–10.53	**0.0057**
Glucose (mmol/L)	106	5.6	2.8–9.2	18	5.5	3.7–56.0	31	5.3	0.9–44.8	0.4256
Bilirubin (µmol/L)	54	3	0.1–27	17	6.7	0.2–588.4	14	5.0	0.1–94.0	**0.0193**
Ammonia (µmol/L)	104	165	15–677	12	58	11–134	10	38	10–70	**<0.0001**
ALT activity (U/L)	101	161	16–2091	101	258	68–1808	30	59	24–286	**<0.0001**
ALP activity (U/L)	103	242	1–7955	21	278	36–2270	30	34	7–107	**<0.0001**
γGT activity (U/L)	39	6	2–21	8	18	0–115	13	5	1–99	**0.0471**

BUN: blood urea nitrogen; ALT: alanine aminotransferase; ALP: alkaline phosphatase; γGT: gamma-glutamyl transferase. Values in bold font are significantly different among the three groups of dogs.

## Data Availability

The anonymized patient data are available from the senior author upon reasonable request.
